# A Pilot Study of Blood Pressure Monitoring After Cardiac Surgery Using a Wearable, Non-invasive Sensor

**DOI:** 10.3389/fmed.2021.693926

**Published:** 2021-08-05

**Authors:** Erez Kachel, Keren Constantini, Dean Nachman, Shemy Carasso, Romi Littman, Arik Eisenkraft, Yftach Gepner

**Affiliations:** ^1^Division of Cardiac Surgery, Cardiovascular Center, Padeh-Poriya Hospital, Tiberias, Israel; ^2^Faculty of Medicine, Bar-Ilan University, Ramat Gan, Israel; ^3^Department of Epidemiology and Preventive Medicine, School of Public Health, Sackler Faculty of Medicine and Sylvan Adams Sports Institute, Tel Aviv University, Tel Aviv, Israel; ^4^Institute for Research in Military Medicine, Faculty of Medicine, The Hebrew University of Jerusalem, Jerusalem, Israel; ^5^Israel Defense Force Medical Corps, Tel Aviv, Israel; ^6^Heart Institute, Hadassah Ein Kerem Medical Center, Jerusalem, Israel; ^7^Biobeat Technologies Ltd., Petah Tikva, Israel

**Keywords:** blood pressure, cardiac surgery, non-invasive sensor, wearable, mean arterial pressure

## Abstract

**Background:** Continuous blood pressure (BP) measurement in intensive care units is based on arterial line (AL) transducers, sometimes associated with clinical complications. Our objective was to evaluate continuous BP measurements obtained from a non-invasive, wireless photoplethysmography (PPG)-based device using two distinct configurations (wristwatch and chest-patch monitors) compared to an AL.

**Methods:** In this prospective evaluation study, comparison of the PPG-based devices to the AL was conducted in 10 patients immediately following cardiac surgery. Pulse rate (PR), systolic BP (SBP), diastolic BP (DBP), and mean arterial pressure (MAP) were recorded using both the AL and the PPG-based devices simultaneously for an average of 432 ± 290 min starting immediately after cardiac surgery. Bland-Altman plots and Pearson's correlations were used to assess the accuracy and degree of agreement between techniques.

**Results:** A total of ~4,000 data points were included in the final analysis. AL measurements for PR, SBP, DBP and MAP were significantly (*p* < 0.001) and strongly correlated with both the wristwatch (*r* = 0.99, *r* = 0.94, *r* = 0.93 and *r* = 0.96, respectively) and the chest-patch (*r* = 0.99, *r* = 0.95, *r* = 0.93 and *r* = 0.95, respectively) monitors. Both configurations showed a marginal bias of <1 mmHg for BP measurements and <1 beat/min for PR [95% limits of agreement −3,3 beat/min; BP measurements: (−6)–(−10), 6–10 mmHg] compared to AL measurements.

**Conclusion:** The PPG-based devices offer a high level of accuracy for cardiac-related parameters compared to an AL in post-cardiac surgery patients. Such devices could provide advanced monitoring capabilities in a variety of clinical settings, including immediate post-operative and intensive care unit settings.

**Clinical Trial Registration:**www.clinicaltrials.gov, NCT03603860.

## Introduction

Post-operative hypotension and hypertension commonly occur specifically after cardiac surgery and are associated with severe outcomes including acute kidney injury, cerebrovascular accidents, myocardial injury and death (1–4). The frequency of post-operative blood pressure (BP) measurement is inversely correlated with hypotension occurrence, and early interventions aimed at adjusting BP are important for improving clinical outcomes (5). A protocol of continuous BP measurement using an arterial line (AL) is currently implemented for 12–24 h in all post-cardiac surgery patients and/or in hemodynamically unstable patients in intensive care units (ICU), operating rooms and post-operative units. Albeit providing accurate and continuous BP measurements, the AL is an invasive method with several potential adverse effects and complications (6). It requires operation by skilled personnel and has a cumbersome setting with monitors, cables and wires connected to the patient (7–10).

Thus, there is a need for alternative methods of continuous BP monitoring that are accurate but also simple to use, non-invasive and ideally wireless. An advanced technology of this sort would allow monitoring of patients who are not routinely monitored due to the technical and logistical shortfalls of traditional measurement techniques such as the AL. Such technology would ultimately assist in early identification of medical emergencies and would enable retrospective investigation of BP when needed, leading to improved patient care (11–13). In recently published work, it was shown that a new reflective photoplethysmography (PPG) device provides accurate measures of BP in a large human cohort and in an animal model of controlled hemorrhagic shock (14, 15). Yet, the device's ability to accurately monitor BP in hospitalized patients in critical care units remains unknown. Thus, the aim of the current study was to assess the level of accuracy of BP measurements between two distinct configurations of the same non-invasive PPG device (Biobeat Technologies LTD, Petah Tikva, Israel) compared to an invasive AL in post-cardiac surgery patients.

## Materials and Methods

### Ethical Considerations

This prospective, comparative clinical trial was registered in www.clinicaltrials.gov (NCT03603860) and was approved by the Institutional Review Board of the Baruch Padeh Medical Center, Poriya, Israel (0077-18-POR). All participants were advised both orally and in writing as to the nature of the experiments and signed an informed consent form before undergoing the surgical procedure.

### Study Population

Eighteen post-cardiac surgery patients (ages 18–81 years; 12 males) were recruited for the study. Monitoring started immediately after the surgical procedure, upon arrival to the Cardiac Surgery Intensive Care Unit (CSICU). The research team only received the monitoring devices' serial numbers without receiving any personal identifiers.

### Study Protocol

**Figure 1:**
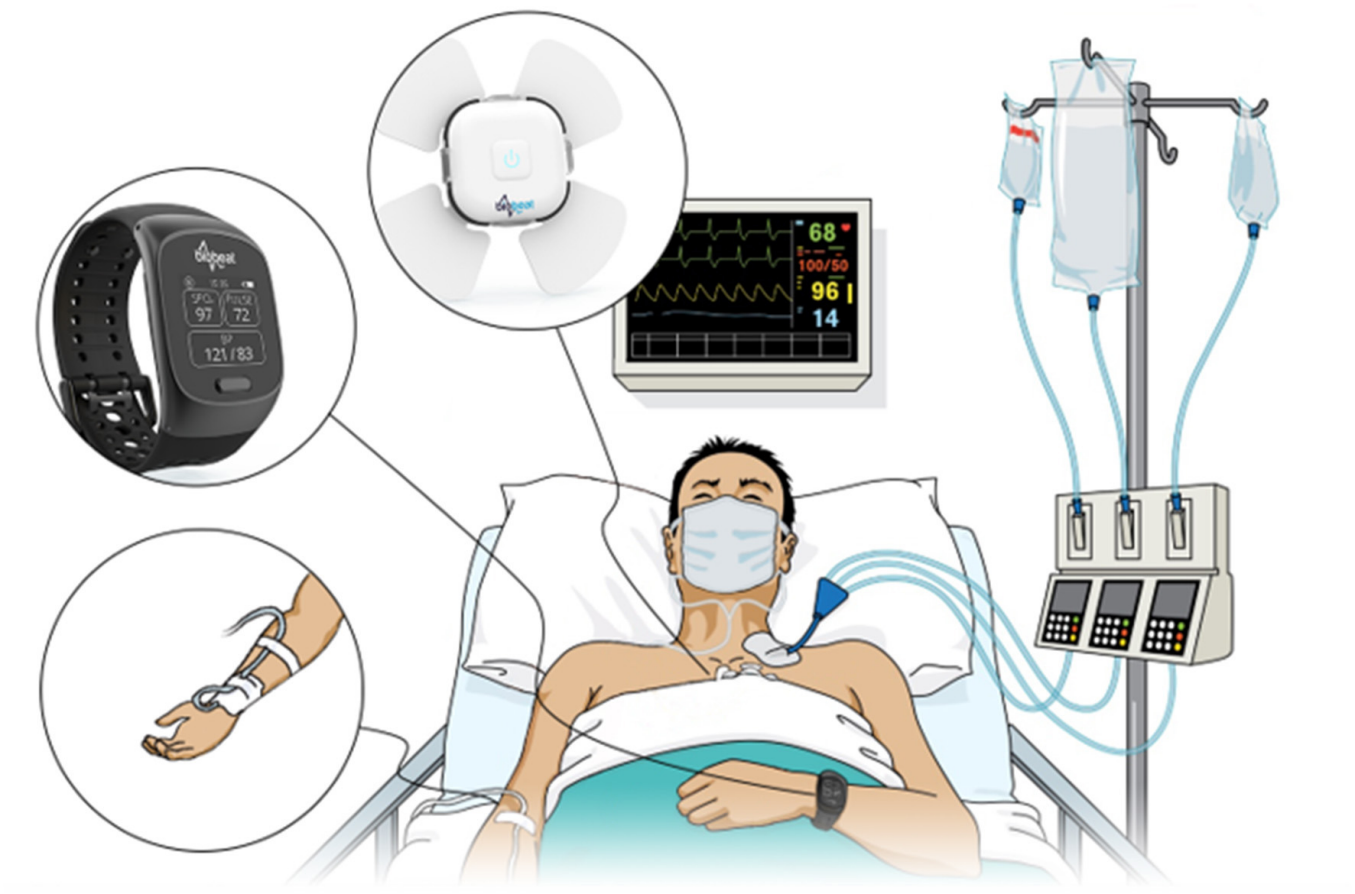
An Arterial line and photoplethysmography-based wristwatch and chest-patch monitors were attached to each subject included in the study.

Patients were transferred to the CSICU immediately after completion of their surgery. In all subjects, an AL (AB-0023 Art-Line™ Kits Single Channel, Biometrix, Gronsveld, The Netherlands, and IntelliVue MX500 Patient Monitor, Philips Medical Systems) was inserted through the radial artery in the operating room prior to surgery, allowing continuous invasive monitoring of BP and pulse rate (PR). The average of the first three BP measurements obtained by the AL was considered as a baseline calibration measurement for the PPG-based devices. From the moment of calibration, the PPG-based devices were attached to the patients – the chest-patch monitors just left to the fresh sternotomy wound and the wristwatch monitors on the arm without the arterial line – and measurements were taken simultaneously, comparing the three devices (i.e., AL, wristwatch monitor, chest-patch monitor) for up to 24 h during the post-operative period (Figure 1). Inclusion criteria included patients arriving immediately after cardiac surgery to the intensive care unit with an AL. Exclusion criteria included refusal to participate, patients with no AL, pregnant women, individuals under the age of 18 years, patients with lack of judgment/mental illness, and patients working in the Baruch Padeh Medical Center.

### The PPG-Based Devices

The device was previously described (14–16). In short, the device is based on reflective PPG technology, in which part of the transmitted light is reflected from the tissue and detected by a photodiode detector positioned near the light source transmitter. The high resolution of the PPG wave combined with advanced algorithms allows analysis using pulse wave transit time (PWTT) combined with Pulse Wave Analysis (PWA). This, in turn, enables tracking of vital signs derived from the pulse contours, including BP changes. The device requires a patient-specific single trimonthly calibration of the PR and BP baseline using an approved cuff-based device. We used both a chest-patch and a wristwatch configuration of the monitoring device to determine the accuracy of each device's measurements separately compared to AL measurements. The measurements are not influenced by arm position with relation to the heart level.

### Data Processing and Analysis

Mean arterial blood pressure (MAP) was calculated as [DBP+⅓·(SBP-DBP)] for each measurement technique. Data obtained from the AL for PR, SBP, DBP and MAP were screened for outliers according to the following criteria: initially, percentage differences (%Δ) between each value and the preceding and succeeding values were calculated. For each variable separately, the average standard deviation (SD) for the sum of preceding and succeeding %Δ was then obtained. Next, the individual data points (%Δ) were screened, and any value for which the percentage difference from both the previous and next values was > ±2SD was eliminated. In total, 132 out of 16,259 (0.8%) outlier values were excluded from the final analysis (PR: *n* = 20/4,305; SBP: *n* = 20/4,018; DBP: *n* = 39/4,018; MAP: *n* = 53/4,018).

### Statistical Analysis

The Kolmogorov-Smirnov and Shapiro-Wilk tests were used to assess normality, as these tests are sensitive to outliers. To define the degree of agreement between each of the non-invasive methods (i.e., chest-patch and wristwatch monitors) and the AL, linear regressions formulas were defined, Pearson's correlation coefficient were calculated, and the hypotheses that the slopes and intercepts are equal to zero were tested. The level of absolute agreement between AL measurements and those obtained from the chest-patch and wristwatch monitors for PR, SBP, DBP and MAP were evaluated using Bland–Altman plots. Results of the Bland–Altman analyses are reported as mean biases ± 95% limits of agreement (LOA). All other results are presented as means ± SD. Statistical analyses were considered significant if *p* < 0.05. Data were analyzed using SPSS 23.0 (SPSS Inc., Chicago, IL, USA). Validity of the non-invasive devices (wristwatch monitor and chest-patch monitor) compared to the gold standard AL was assessed as the devices' ability to correctly identify extreme values (SBP <90 or > 160 mmHg, DBP <60 or > 100 mmHg and MAP <65 or > 160 mmHg) (17, 18) using sensitivity, specificity and positive and negative predictive values.

## Results

Of the 18 patients initially recruited, eight were excluded due to non-continuous monitoring of the AL and thus inability to compare these subjects' data between the wristwatch/chest-patch monitors and AL. Demographic data and characteristics for the remaining ten subjects (60 ± 15 years; weight: 76.5 ± 9.1 kg; height: 1.69 ± 0.06 m) including the type of surgery they underwent are presented in Table 1. Patients were monitored for an average time of 549 ± 251 min. For two subjects, data was obtained every 10 min due to technical limitations of the AL (63 measurement time-points in one patient and 82 in the other), while for eight subjects, measurements were recorded every minute with the average number of samples (time points) being 522 ± 249 (18).

**Table 1:** Patient characteristics.

**Subject number**	**Gender**	**Age (years)**	**Weight (Kg)**	**Height (cm)**	**Area of origin**	**Fitzpatrick scale**	**Surgical procedure**
1	M	60	100	175	Western Europe	Type l	CABG
2	F	75	69	165	Western Europe	Type lll	MVR+TVR
3	F	45	71	170	Middle East	Type lll	MVR
4	F	45	75	160	Middle East	Type lV	CABG
5	M	60	75	165	Middle East	Type V	CABG
6	M	81	75	165	Western Europe	Type ll	AVR+TVR+AA
7	M	35	83	180	Eastern Europe	Type lll	CABG
8	F	76	75	165	Middle East	Type lV	CABG
9	M	64	70	165	Middle East	Type V	CABG
10	M	57	72	175	Western Europe	Type ll	MVR

*AVR, aortic valve replacement; CABG, coronary artery bypass graft; MVR, mitral valve replacement; TVR, tricuspid valve replacement; AA, aortic aneurism repair. The Fitzpatrick Scale is based on Fitzpatrick et al. (19)*.

There were no adverse events while using the PPG-based devices. The number of comparisons for each variable and mean ± SD values for each variable per device are presented in Table 2. None of the patients received catecholamines during the study. There were no AL-related complications during the study.

**Table 2:** Number of comparisons for each variable and mean ± SD values for each variable per device.

**Variable**	***n***	**Mean±** **SD**		
		**Invasive**	**Watch**	**Patch**
HR (beats/min^−1^)	4,285	78 ± 10	78 ± 10	78 ± 10
SBP (mmHg)	3,998	120 ± 14	120 ± 15	120 ± 15
DBP (mmHg)	3,979	61 ± 8	61 ± 9	61 ± 9
MAP (mmHg)	3,970	81 ± 9	81 ± 9	81 ± 9

*HR, Heart rate; SBP, Systolic blood pressure (BP); DBP, Diastolic BP; MAP, mean BP*.

### Degree of Agreement

**Figure 2:**
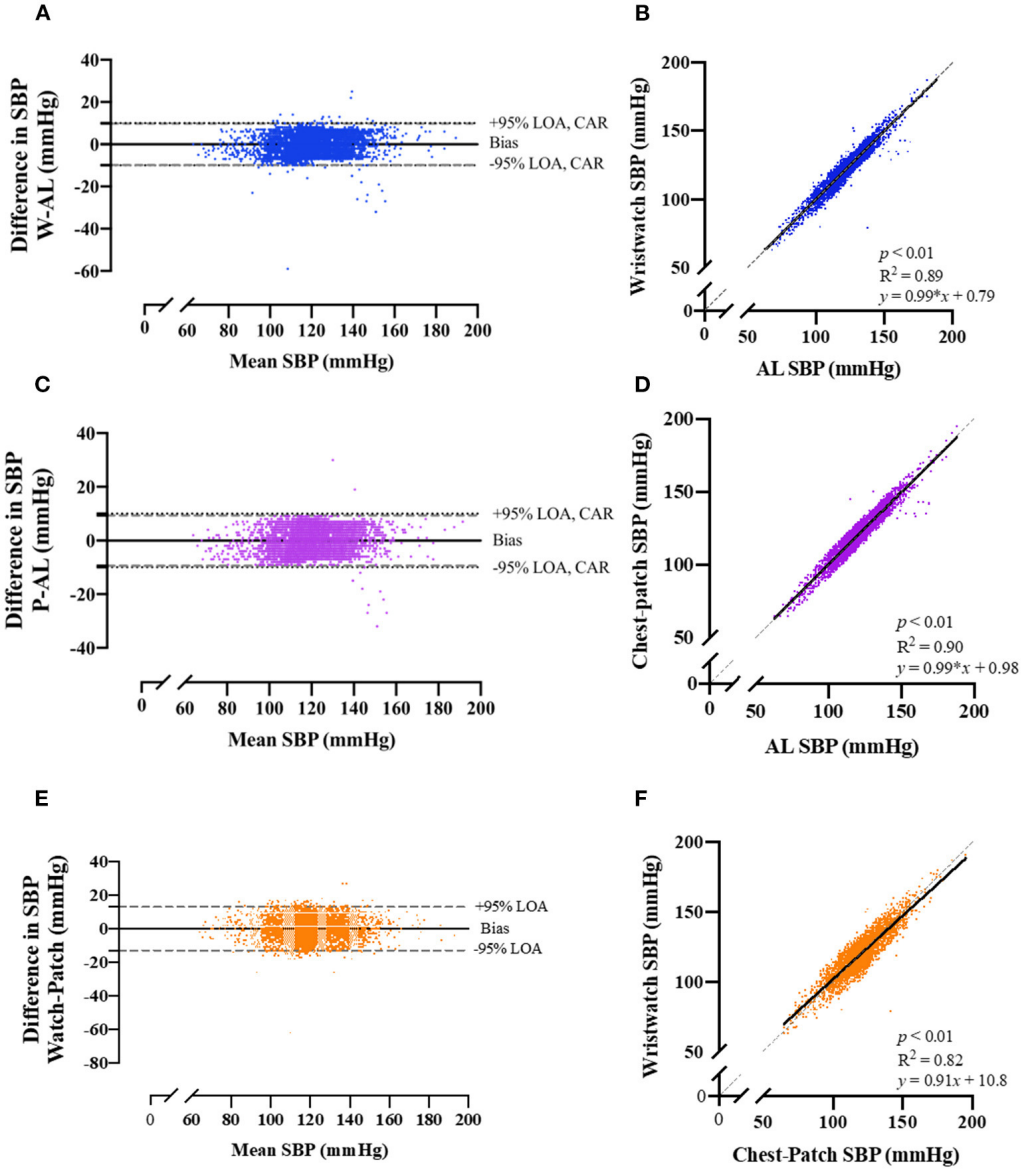
Bland-Altman plots of the relationship and limits of agreement between the photoplethysmography-based devices' non-invasive systolic blood pressure measurements and the arterial line's invasive measurements. Bland-Altman plots (left panels) and Pearson's correlations (right panels) are shown for wristwatch and arterial line **(A,B)**, chest-patch and arterial line **(C,D)**, and wristwatch and chest-patch **(E,F)**. In the right panels, the solid line is the best fit linear regression and the dash line is the line of identity. In the left panels, the solid horizontal line represents the mean difference between the two measurements (bias), the dash horizontal lines represent the 95% limits of agreement (LOA), and the dotted lines represent the clinically accepted range (CAR). W, wristwatch configuration; CP, chest patch configuration; SBP, systolic blood pressure; AL, arterial line.

**Figure 3:**
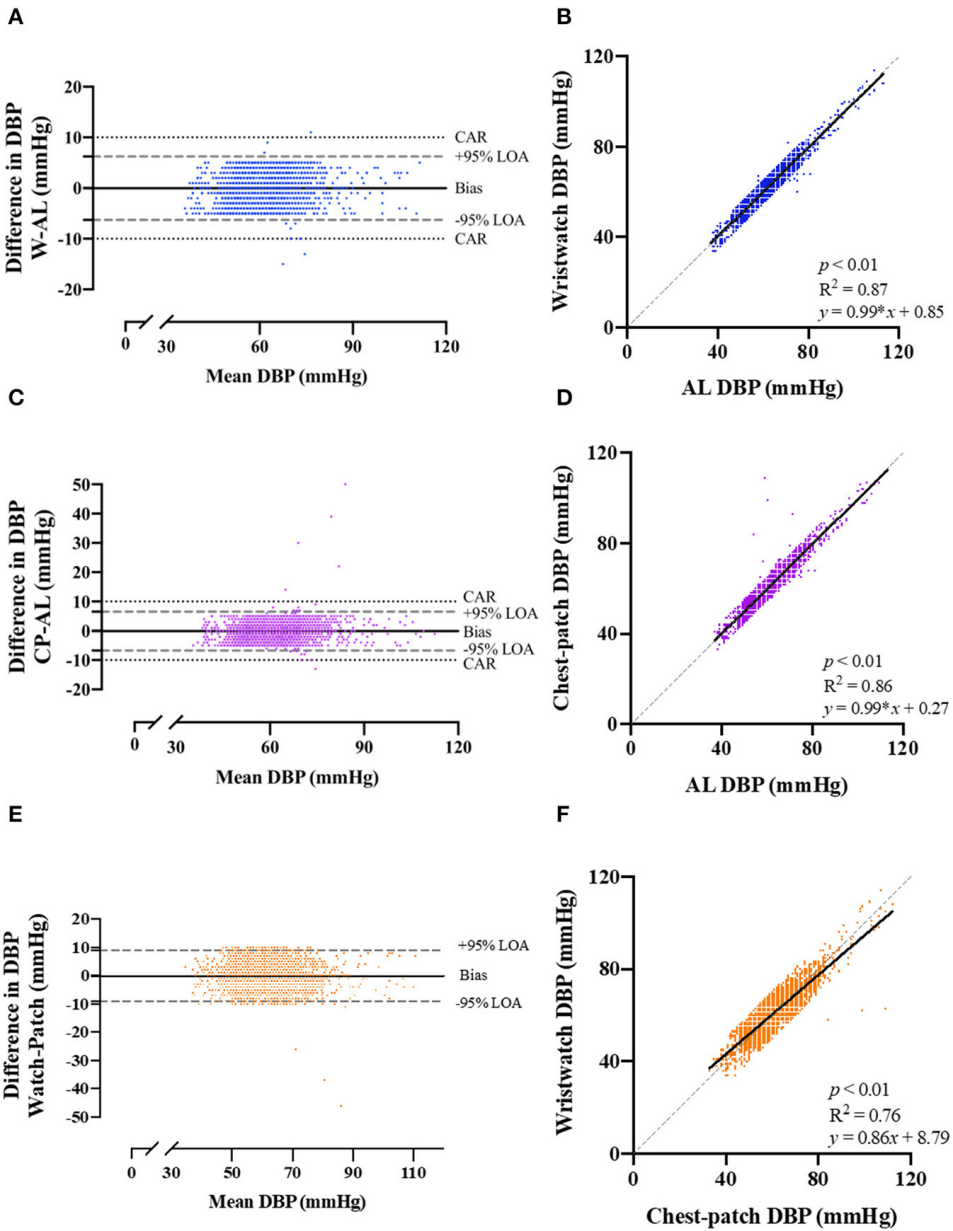
Bland-Altman plots of the relationship and limits of agreement between the photoplethysmography-based devices' non-invasive diastolic blood pressure measurements and the arterial line's invasive measurements. Bland-Altman plots (left panels) and Pearson's correlations (right panels) are shown for wristwatch and arterial line **(A,B)**, chest-patch and arterial line **(C,D)**, and wristwatch and chest-patch **(E,F)**. In the right panels, the solid line is the best fit linear regression and the dash line is the line of identity. In the left panels, the solid horizontal line represents the mean difference between the two measurements (bias), the dash horizontal lines represent the 95% limits of agreement (LOA), and the dotted lines represent the clinically accepted range (CAR). W, wristwatch configuration; CP, chest patch configuration; DBP, diastolic blood pressure; AL, arterial line.

Pearson correlations and Bland-Altman plots with 95% LOA for each of the BP variables are presented in Figures 2, 3, and Supplementary Figure 1. SBP, DBP and MAP values obtained from the wristwatch and chest-patch monitors demonstrated a small bias (<1 mmHg) and relatively narrow LOA (wristwatch: SBP: −10,10 mmHg, DBP: −6,6 mmHg, MAP: −5,5 mmHg; chest-patch monitor: SBP: −9,9 mmHg, DBP: −7,7 mmHg, MAP: −6,6 mmHg) compared to AL. This strong agreement was also found for PR, where the wristwatch and chest-patch monitors' values had a bias of <1 beat/min and the LOA were −3,3 beat/min compared to AL (Supplementary Figure 2).

Percent of observations obtained within the clinical definition of 5, 10 and 15 mmHg between the wristwatch and chest-patch as compared to AL are shown in Table 3.

**Table 3:** Percent of observations obtained from the wristwatch and chest-patch configurations of a wearable, non-invasive sensor that were within 5, 10, and 15 mmHg of those obtained by an arterial line.

		**≤5**	**≤10**	**≤15**
Watch–AL	SBP	68.9%	99.1%	99.6%
	DBP	99.7%	99.9%	100.0%
Patch–AL	SBP	70.1%	99.6%	99.7%
	DBP	99.2%	99.8%	99.9%

*AL, Arterial line; SBP, Systolic blood pressure (BP); DBP, Diastolic BP. Differences between devices were calculated as mean absolute difference*.

As demonstrated in Figures 2, 3, and Supplementary Figure 1, AL measurements for SBP, DBP and MAP were significantly (*p* < 0.001) and strongly correlated with the wristwatch (SBP: *r* = 0.94; DBP: *r* = 0.93; MAP: *r* = 0.96) and with the chest-patch (SBP: *r* = 0.95; DBP: *r* = 0.93; MAP: *r* = 0.95) monitors. Lastly, PR values obtained by AL were significantly (*p* < 0.001) and strongly correlated with the wristwatch (*r* = 0.99) and with the chest-patch (*r* = 0.99) monitors (Supplementary Figure 2).

Next, we compared the reliability between the wristwatch to the chest-patch (Figures 2, 3, and Supplementary Figures 1, 2). There was a small (<1 mmHg) bias for all BP measures and the LOA's were as follows; SBP: −13,13 mmHg, DBP: −9,9 mmHg, MAP: −7,7 mmHg. BP measurements obtained from the wristwatch and chest-patch configurations were significantly (*p* < 0.001) and strongly correlated (*r* = 0.91, 0.87 and 0.92 for SBP, DBP and MAP, respectively). Lastly, we present the distribution and the median of each vital as compared to the AL (Supplementary Figure 3).

## Discussion

In this observational study, we have shown that the tested non-invasive, PPG-based devices provide an accurate assessment of SBP, DBP, MAP and PR compared to the invasive, gold-standard measurements obtained using an AL in post-cardiac surgery patients. Specifically, the two configurations of the device, wristwatch and chest-patch monitors alike, each showed a strong level of agreement with AL measurements, as evident by strong correlations, marginal bias and narrow LOA for SBP, DBP, MAP and PR. With an AL requiring trained personnel for placement and having an inherent risk of infection and damage to arteries and nerves associated with it (6, 10), there is much need in clinical settings for safe and accurate measurement techniques. The devices tested in this study provide accurate hemodynamic measurements non-invasively and in a safer manner. This has important implications for clinical settings, as such measures are crucial for improved prognosis and mortality in high-risk patients (5). Additional advantages of the devices are that they are wireless, cuff-less and easy to operate, thus overcoming two main drawbacks of the AL: (1) its cumbersome setting, which includes cables and monitors placed on and around patients who also have numerous other cables and machinery surrounding them, and (2) the need for frequent calibration. Implementation of such devices opens a wide range of cases/scenarios in which they can be used in hospitalized patients, including non-cardiac patients, thus allowing an improved monitoring capability in patients that usually do not get an AL, or after an AL has been removed. Ultimately, this method of continuous, non-invasive monitoring will provide longer periods of monitoring than what is currently implemented.

Post-operative hypotension is common, and often goes undetected by routine intermittent vital sign assessments (20). Moreover, it is strongly associated with morbidity and mortality even several days after the surgical intervention. Continuous BP measurement allows early detection and intervention in cases of post-operative hypotension (20), and therefore, any technique aiming to improve patient outcomes should provide continuous readings of key physiological parameters such as BP. Hemodynamic monitoring by non-invasive means could reduce the morbidity seen with invasive devices such as the AL, which despite being regarded as the gold standard measurement technique within intensive care units, is associated with a high prevalence of infection and local vascular damage (8).

PPG technology has been widely used in medical care in recent years. This technology provides easy patient monitoring without the need for extensive training of health care workers, while allowing reliable, accurate, and safer monitoring of hospitalized patients who may be in a critical, unstable clinical condition. Several studies examined the accuracy of wearable PPG-based sensors compared with electrocardiograms, pulse oximeters, and chest straps in various conditions. These studies concluded with mixed findings as a result of inappropriate comparison methods and lack of reproducibility due to the use of different versions of the software, to name a few (21–26). Previous studies have shown the potential of tracking changes in BP in clinical settings using the non-invasive Pulse Wave Transit Time method (27–29). However, validation studies are needed before implementation of such devices in clinical settings would be possible (30). Similar to the above-mentioned method, the devices tested in this study track changes using Pulse Wave Transit Time, recently shown to provide accurate BP measurements compared to the widely-used cuff-based manometry (14) and accurately track changes in cardiac output and BP during unstable hemodynamic conditions in a swine model of controlled hemorrhagic shock (15).

**Figure 4:**
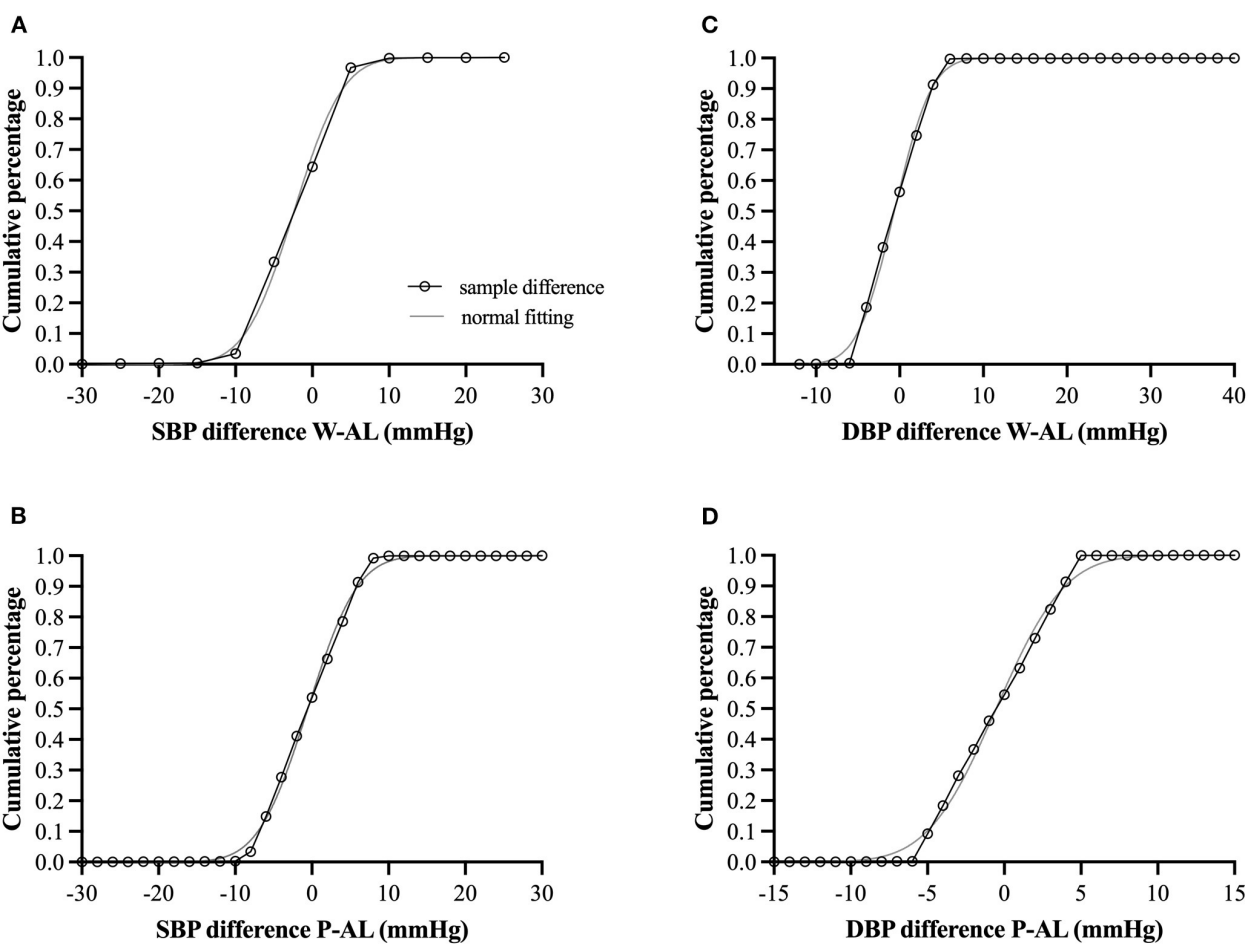
**(A–D)** Cumulative percentage difference (open circles connected with black solid line) for the observed absolute differences between arterial line (AL) and the photoplethysmography-based sensors with corresponding hypothesized normal cumulative distribution (gray solid line). W, wristwatch configuration; P, chest-patch configuration; DBP, diastolic blood pressure; SBP, diastolic blood pressure; AL, arterial line. For all comparisons, the goodness of fit (R2) was > 0.99 for both SBP and DBP.

Criteria for cuff-less monitors such as the PPG-based sensor have yet to reach widespread, general acceptance. Based on the European Society of Hypertension's (ESH) International Protocol for the validation of BP measuring devices (31), *post-hoc* analysis revealed as many as 69–70% of the SBP values and >99% of the DBP values obtained from the PPG-based devices (both wristwatch and chest-patch monitors) were within ±5 mmHg of those recorded by AL. These percentages also classify the PPG-based sensor as *Grade A* according to the British Hypertension Society (31) and IEEE Standard for Wearable, Cuffless Blood Pressure Measuring Devices (32) (Table 3; Figure 4). As can be seen in Figure 4, the goodness of fit (R^2^) for the observed absolute differences between AL and the PPG-based sensors was > 0.99 for both SBP and DBP.

Furthermore, we have shown high levels of agreement (Pearson's correlation >0.93 for SBP, DBP and MAP for both wristwatch and chest-patch monitors; Figures 2, 3, and Supplementary Figure 1), and relatively narrow 95% LOAs (: (−6)–(−10), 6–10 mmHg) between the wearables and AL measurements, suggesting the PPG-based devices can accurately and reliably measure SBP and DBP.

An important capacity for a clinical device is its ability to track hemodynamic changes and to detect instability, including correct identification of severe cases and/or extreme values (i.e., positive and negative predictive values, sensitivity, and specificity). For this reason, we sought to evaluate the PPG-based sensor following pre-established, clinically significant extreme values for BP measures. However, although our cohort included cardiac patients immediately post-surgery, there were not many cases of hypotension (as determined by SBP <90 mmHg, DBP <60 mmHg or MAP <65 mmHg) or hypertension (as determined by SBP > 160 mmHg or DBP > 100 mmHg). In fact, apart from DBP <60 mmHg where 1,609 extreme cases were identified (40%), for all other criteria <3% of values were identified as extreme values, technically limiting our ability to obtain meaningful and reliable predictive values and sensitivity/specificity measures.

Since the two configurations of the device tested in our study measure vital signs in different locations, we chose to utilize both in order to demonstrate that accurate measurements can be obtained independently of the site of measurement (i.e., wrist or chest). In the hospital environment, a single use disposable chest-patch monitor is estimated to further reduce infection transmission and the potential for error between patients than a wristwatch that is used on several patients. In contrast, a wristwatch might be more optimal for long-term home monitoring of chronic patients. Beyond in-hospital intensive care and low acuity patients, PPG technology could enable better monitoring, triage and treatment in prehospital scenarios as well. Moreover, the current severe acute respiratory syndrome coronavirus-2 (SARS-CoV-2) pandemic highlights the need for frequent, non-invasive and wireless devices for remote monitoring of isolated patients, requiring minimal direct contact of medical staff, while maintaining and not impairing the quality of care (33).

It should be mentioned that during the study, patients were relatively stable, precluding us from seeing many cases of hemodynamic instability despite their being immediately post-operative. Still, short periods of hemodynamic instability were observed, in which we found a high level of agreement between the devices. Although this study did not include a large sample, each individual had multiple (~400) measurement points, and the results show high levels of agreement and strong correlations between devices across a wide range of BP values (SBP: 63–188 mmHg, DBP: 37–113 mmHg, MAP: 47–125 mmHg). Moreover, seeing as one of the strengths of the tested devices is their ability to provide accurate and frequent monitoring, allowing to include a small sample of 10 subjects in this study with high confidence of obtaining large amounts of reliable data.

Since the PPG-based device is dependent on pulse wave, it will not suit certain patient populations, such as patients with left ventricular assist device and intra-operative monitoring during the use of cardio-pulmonary bypass. However, post-surgery patients might benefit from a non-invasive sensor that will replace an invasive in-dwelling monitoring device. Beyond the monitoring capabilities of an AL, it is worth mentioning that the purpose using this technique specifically in patients undergoing cardiac surgery goes beyond blood pressure measurement, and blood samples are needed throughout surgery to assess blood gases, coagulation parameters, and more. This feature is not included in the PPG-based platform and might influence the decision whether to use an AL or a non-invasive device.

## Conclusions

There is a clear need for a reliable, non-invasive technology capable of advanced hemodynamic monitoring that would overcome the limitations of the currently and frequently used AL technique. In this observational clinical study, we report that measurements obtained from the two configurations of a novel PPG-based device offer a high level of accuracy compared to the existing standard invasive technique in post-cardiac surgery patients. Most importantly, this PPG-based technology enables continuous, non-invasive remote patient monitoring and timely focused care, which may minimize morbidity and mortality without compromising measurement accuracy. Future studies should focus on the advanced monitoring capabilities of these devices in a variety of clinical settings, from immediate hospital-based post-operative and intensive care units to post-hospital and ambulatory settings.

## Data Availability Statement

The raw data supporting the conclusions of this article will be made available by the authors, without undue reservation.

## Ethics Statement

The studies involving human participants were reviewed and approved by Institutional Review Board of the Baruch Padeh Medical Center, Poriya, Israel (0077-18-POR). The patients/participants provided their written informed consent to participate in this study.

## Author Contributions

EK, DN, SC, and AE: study conception and design. EK, DN, SC, and RL: data collection. KC, DN, AE, and YG: analysis and interpretation of results. KC and YG: draft manuscript preparation. All authors reviewed the results and approved the final version of the manuscript.

## Conflict of Interest

YG was advisor in the physiology board of Biobeat Technologies Ltd. AE and RL were employees at Biobeat Technologies Ltd. The remaining authors declare that the research was conducted in the absence of any commercial or financial relationships that could be construed as a potential conflict of interest.

## Publisher's Note

All claims expressed in this article are solely those of the authors and do not necessarily represent those of their affiliated organizations, or those of the publisher, the editors and the reviewers. Any product that may be evaluated in this article, or claim that may be made by its manufacturer, is not guaranteed or endorsed by the publisher.

## Supplementary Material

Supplementary Figure 1 Bland-Altman plots of the relationship and limits of agreement between the photoplethysmography-based devices' non-invasive mean arterial pressure measurements and the arterial line's invasive measurements. Bland-Altman plots (left panels) and Pearson's correlations (right panels) are shown for wristwatch and arterial line [panels **(a,b)**], chest-patch and arterial line [panels **(c,d)**], and wristwatch and chest-patch [panels **(e,f)**]. In the right panels, the solid line is the best fit linear regression and the dash line is the line of identity. In the left panels, the solid horizontal line represents the mean difference between the two measurements (bias), the dash horizontal lines represent the 95% limits of agreement (LOA), and the dotted lines represent the clinically accepted range (CAR). MAP, mean arterial pressure; W, wristwatch configuration; CP, chest patch configuration; AL, arterial line.

Supplementary Figure 2 Bland-Altman plots of the relationship and limits of agreement between the photoplethysmography-based devices' non-invasive pulse rate measurements and the arterial line's invasive measurements. Bland-Altman plots (left panels) and Pearson's correlations (right panels) are shown for wristwatch and arterial line **(a,b)**, chest-patch and arterial line **(c,d)**, and wristwatch and chest-patch **(e,f)**. In the right panels, the solid line is the best fit linear regression and the dash line is the line of identity. In the left panels, the solid horizontal line represents the mean difference between the two measurements (bias), the dash horizontal lines represent the 95% limits of agreement (LOA), and the dotted lines represent the clinically accepted range (CAR). PR, pulse rate; AL, arterial line; W, wristwatch configuration; CP, chest patch configuration.

Supplementary Figure 3 Individual data for pulse rate, systolic blood pressure and diastolic blood pressure after cardiac surgery determined using an arterial line and a wearable, non-invasive sensor in two configurations: wristwatch and chest-patch. Red horizonal lines represent the median of each vital. PR, pulse rate; SBP, systolic blood pressure; DBP, diastolic blood pressure.
