# Supplementary figures and images for: A Pilot Study of Blood Pressure Monitoring After Cardiac Surgery Using a Wearable, Non-invasive Sensor
Source: Front Med (Lausanne). 2021 Aug 5;8:693926. doi: 10.3389/fmed.2021.693926 (PMC8375406; doi:10.3389/fmed.2021.693926)

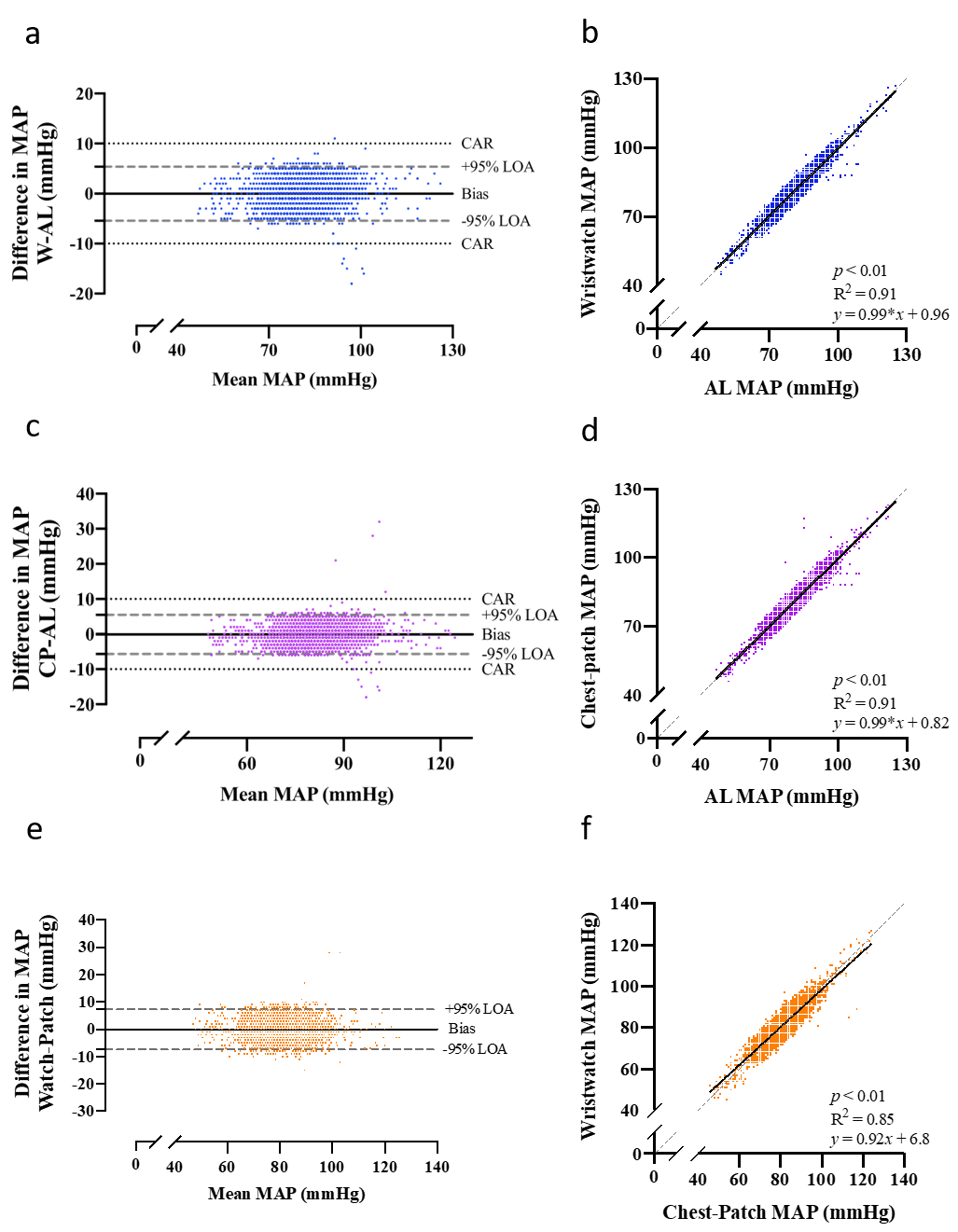

Supplement: Supplementary Figure 1 — Bland-Altman plots of the relationship and limits of agreement between the photoplethysmography-based devices' non-invasive mean arterial pressure measurements and the arterial line's invasive measurements. Bland-Altman plots (left panels) and Pearson's correlations (right panels) are shown for wristwatch and arterial line [panels (a,b)], chest-patch and arterial line [panels (c,d)], and wristwatch and chest-patch [panels (e,f)]. In the right panels, the solid line is the best fit linear regression and the dash line is the line of identity. In the left panels, the solid horizontal line represents the mean difference between the two measurements (bias), the dash horizontal lines represent the 95% limits of agreement (LOA), and the dotted lines represent the clinically accepted range (CAR). MAP, mean arterial pressure; W, wristwatch configuration; CP, chest patch configuration; AL, arterial line. [file Image_1.TIF]

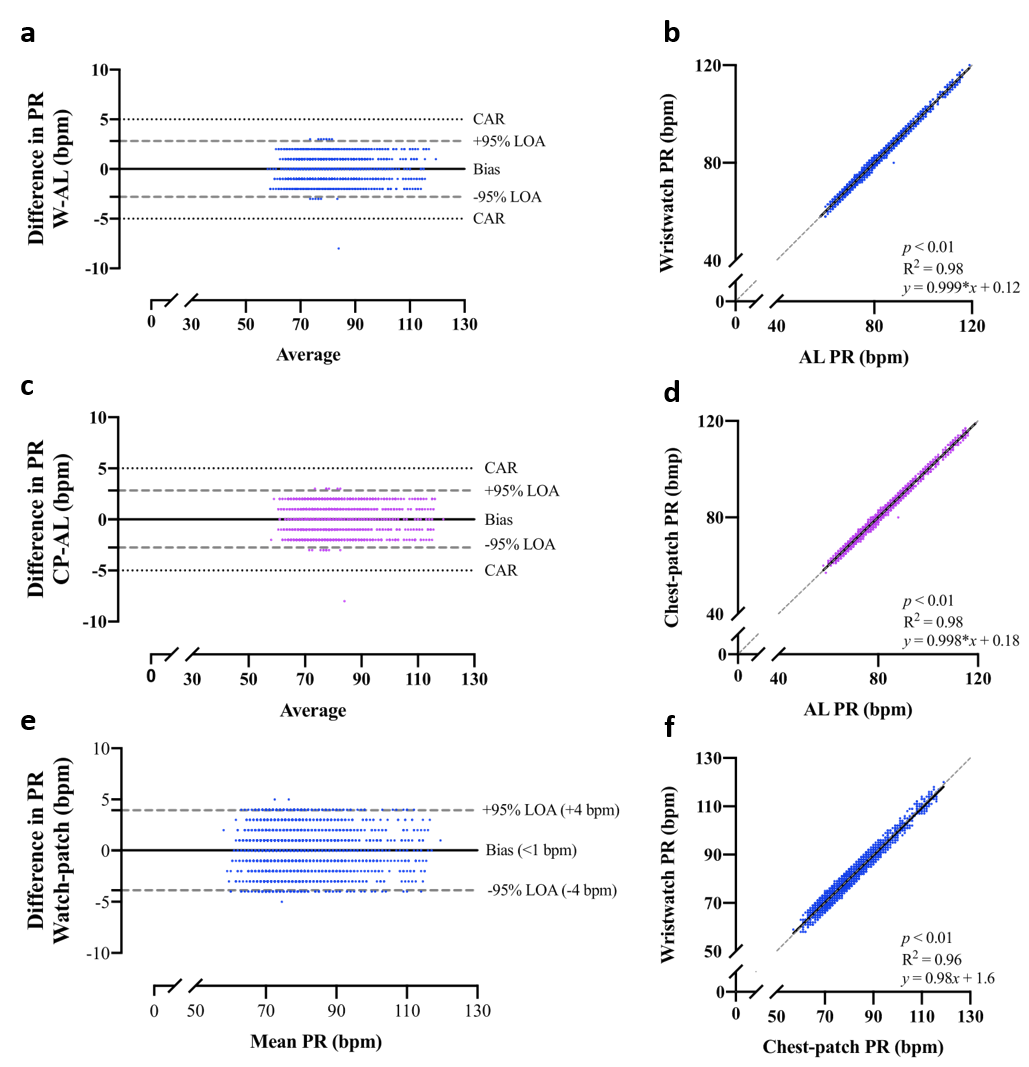

Supplement: Supplementary Figure 2 — Bland-Altman plots of the relationship and limits of agreement between the photoplethysmography-based devices' non-invasive pulse rate measurements and the arterial line's invasive measurements. Bland-Altman plots (left panels) and Pearson's correlations (right panels) are shown for wristwatch and arterial line (a,b), chest-patch and arterial line (c,d), and wristwatch and chest-patch (e,f). In the right panels, the solid line is the best fit linear regression and the dash line is the line of identity. In the left panels, the solid horizontal line represents the mean difference between the two measurements (bias), the dash horizontal lines represent the 95% limits of agreement (LOA), and the dotted lines represent the clinically accepted range (CAR). PR, pulse rate; AL, arterial line; W, wristwatch configuration; CP, chest patch configuration. [file Image_2.TIF]

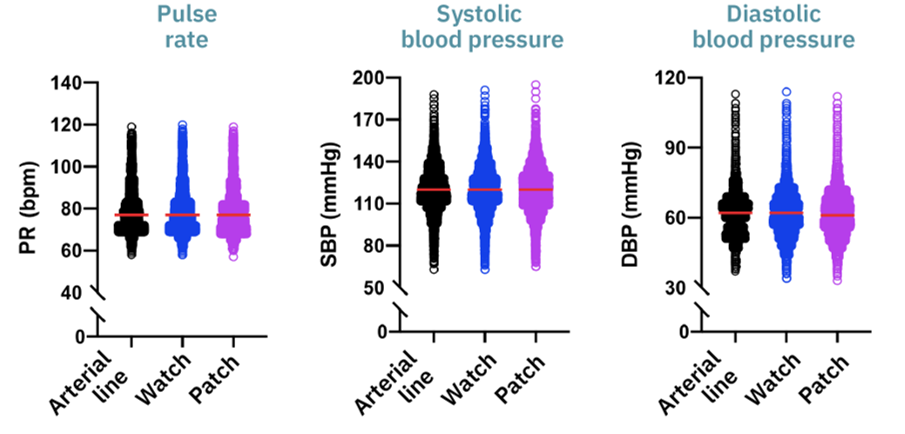

Supplement: Supplementary Figure 3 — Individual data for pulse rate, systolic blood pressure and diastolic blood pressure after cardiac surgery determined using an arterial line and a wearable, non-invasive sensor in two configurations: wristwatch and chest-patch. Red horizonal lines represent the median of each vital. PR, pulse rate; SBP, systolic blood pressure; DBP, diastolic blood pressure. [file Image_3.TIF]
